# Facts and Observations on Quickening

**Published:** 1812-06

**Authors:** 


THE
Medical and Physical Journal.

6 OF VOL. XXVII.] JUNE, 1812. [NO. 160.

*For the Medical and Physical Journal.*

### FACTS *and* OBSERVATIONS *on* QUICKENING.

Præterea, si inmortalis natura animai
Constat, et in corpus nascentibus insinuatur.—*Lucretius*, lib. 3.

OPINIONS and investigations which respect the nature and mode of existence of that immortal principle called the *Soul*, prior to its taking possession of its corporeal tenement, have never established any other fact so firmly, as that the human intellect possesses the property of perplexing a subject, to a most extravagant degree, by the aid of learning and philosophy. The notion of the pre-existence of the human soul, consisting, according to Pythagoras and Plato, of a divine idea united to a portion of the *anima mundi*, has spread far and wide. In Greece the principle of the metempsychosis took full possession of the most refined and refining philosophers; and the reminiscence* of Plato resounded in the groves of the academy, awakening the imagination to the recognition of events which had passed in former modes of existence, when the soul, travelling from being to being, now incited a hero at the Trojan war, now flushed the cheek of some fair Helen, flew in birds, swam in fishes, or crawled, in penal degradation, in some horrid reptile. Absurd, or devoid, at least, of sober certainty, as the metempsychosis must be, we find that both the savage and the sage, at one period of the world, admitted its validity. Among the celtic† nations in the

* Intellection is the operation of the understanding contemplating intelligible forms, or ideas. It is twofold: the first, that of the soul contemplating ideas before it descends into the body; the other, that which it exercises after it is immersed in the body. The first kind of knowledge consists in the recollection of those things which the mind had known in its pre-existent state, and differs from memory in the object; memory being employed upon sensible things, reminiscence upon things purely intelligible.—*Vide Brucker's Historia Critica Philosophiæ.*

† A passage in Lucan ascribes the doctrine of transmigration to the Northern nations.

——————— Vobis auctoribus, umbræ

Non tacitus Erebi cedes, ditisque profundi

Pallida regna petunt: regit idem spiritus artus

Orbe alio: longæ, canitis si cognita, vitæ

Mors media est. *Phars.* lib. 1. v. 454.

religions of Hindu, and the oriental tribes both of the continent and islands of the Indian ocean, we meet with this opinion.*

Little suited as these observations may at first seem to a Medical Journal, they are not totally irrelevant, because this notion of the separate existence of the soul, and its capability or property of being infused into bodies at particular periods, countenanced an hypothesis that professed to explain a natural fact occurring, under very interesting circumstances, to the human female. If it were admitted that the principle of animation, or the soul, was added to the embryo at any distinct period of gestation, posterior to its first formation, it might account for the peculiar sensation which is experienced by women about the sixteenth week of pregnancy; and which sudden infusion not only the vague and visionary theorist believed, but the sober judge on the bench, and the grave doctor in the professional chair, taught or enforced. When the professor mixed up his physiology with the abstract metaphysical notion of the pre-existent state of the soul, and its application to the corporeal fabric at some particular period of utero gestation, his mistake was

* In the institutes of *Menu*, there is a scale of the penalties to which the immortal soul is doomed. "For sinful acts, that are mostly corporeal, a man shall assume, after death, a vegetable or mineral form: for such acts, mostly verbal, the form of a bird or beast: for sinful acts, mostly mental, the lowest of human conditions." The Jewish *Cabbala* holds that all souls were produced at once, and pre-existed in Adam; and that every human soul has two guardian angels, produced by emanation, at the time of the production of souls. The cabbalistic doctrine is minute and elaborate in its investigation of the human soul, which it describes as consisting of four parts: *Nephesh*, or the principle of vitality; *Ruach*, or the principle of motion; *Neschamah*, or the power of intelligence; and *Jechidah*, or the divine principle. The old doctrine of *Quickening* might have raised an implacable war between *Nephesh* and *Ruach*. Not very remote from the doctrine of the *Cabbala* was that opinion of Plato which makes the soul consist of three parts: the first, the seat of intelligence; the second, of passion; the third, of appetite. The Egyptians, according to Herodotus and Diogenes Laertius, believe, that, when the body decays, the soul passes into some other animal, which is then born; and that, after it has made the circuit of beasts, birds, and fishes, through a period of 3000 years, it again becomes an inhabitant of a human body. To collect and detail the opinions of Aristotle, Socrates, Pythagoras, Empedocles, Democritus, Heraclitus, Epicurus, Strabo, Dicearchus, &c. &c. on this abstruse subject, would hardly reward the labour.

"Much of the soul they talk, but all awry."—*Milt. P. R.* iv. 313.

no no farther important, perhaps, than as it led his pupils aside from the plain path of truth, into the tangled wilderness of error, and suppressed the still small voice of Nature with the obstreperous jargon of the schools; but, when the judge pronounced on the innocence or criminality of actions on this hypothesis, truth was not only violated, but society sustained an injury.

As the opinion above alluded to has been, if it is not even now, very generally held, it may not be quite improper, to state what is, probably, the true nature of the sensation called QUICKENING.

I need not cite evidence to prove that the ancient philosophers and physicians, and the earliest of the modern professors of the obstetric art, believed that the peculiar sensation of Quickening, as it is erroneously called, occurred when the fœtus received the principle of vitality, or became animated; and that the specific feeling in the mother was produced by its first motions. Courts of justice recognised this hypothesis also, as a principle by which the degree of criminality in cases of *abortus procuratus* were to be determined. If these were the opinions of times long past away with their errors and their delusions, how stands the question now? This will be answered by allowing the grave and learned leaders of the *ars obstetrica* to tell their own tale.

Dr. Denman (Introduction to the Practice of Midwifery, 5th edit. 1805,) says, "By the term *Quickening* is understood *the first sensation which the mother has of the motion of the child* which she has conceived. This happens at different periods of pregnancy, from the tenth to the twenty-fifth week, but most commonly about the sixteenth after conception; yet the motion of the child is in *some women so obscure*, or such little attention is paid to it, that it is not perceived or regarded, and in others so indistinct as to be confounded with various other sensations. It is not unusual for women to have a few drops of blood discharged from the vagina at the time of *quickening;* but the symptoms which attend are generally such as are occasioned by surprise or agitation from any other cause, as fainting, or some hysteric affection." Of the changes which succeed immediately upon the sensation of quickening he speaks with more precision;* and, if such a fact did not frequently occur, it would be

* These changes have no connection whatever with the first motions of the child, but are altogether produced by the alteration of position in the uterus. "The changes which follow quickening, (the be surprising that, when so near the truth, he had not distinctly seen what the cause of the sensation of *quickening* really was. But it is left as much in the dark, as it was at any former period. The changes consequent on quickening are not quickening itself; they are the effect of this alteration in the female œconomy, whatever it may be.

Professor Hamilton (*Outlines*, 5th edit. 1806) has almost stepped upon the true nature of this peculiar sensation. "Faintings," he says, "seldom occur," (that is, during pregnancy,) "except about the time of *quickening*. They seem to arise from the sudden change of position of the uterus, emerging from its close confinement within the bony parieties of the pelvis, and from the irritation *communicated by the child's motion*." He does not, however, intend here to explain what *quickening* itself is, but looks to one of its phenomena, deliquium; and he hangs upon the old opinion of the child's motion, as an efficient cause of the mother's sensation. So little did he, probably, understand of the real nature of this incident in 1806, that he must be considered as having left it in absolute darkness; though he approached to the truth, the veil was not removed. In the year 1811, his countryman, John Burns, who, when writing on the "Principles of Midwifery," could not but have seen the professor's "Outlines," had no glimpse of the true nature of *quickening*: "In some cases, very little disturbance is produced," (that is, by the impregnated state,) he says, "and the woman is not certain of her condition, until the child quickens, which happens about the fourth or fifth month of pregnancy, in a few

---

(the doctor says,) have been attributed to various causes: by some it has been conjectured that the child then acquired a new mode of existence; or that it was arrived at a size to be able to dispense with the menstruous blood, &c. Others have believed, that the changes ought to be assigned merely to the enlargement of the uterus, increased *by the growth of the ovum* to such a size, that it was supported above the brim of the pelvis; by which means all the inconveniences which arose from the dragging or subsistence of the uterus in the vagina were removed: and this seems to be the true reason." But these changes, as before observed, have no manner of connection with quickening, as referred to the first motions of the fœtus; and the confounding under this term the first motions of the child, with the specific sensation occasioned by the sudden change of the womb's position, and then explaining the alterations in the woman's health, by referring them to quickening, or the first perceived motions of the child, are so illogical, as hardly to have a trace of the true fact. Neither the specific sensation, nor the ceasing of the irritability, of early pregnancy, depend, in any degree, on the first motions of the fœtus.

instances instances at the end of the third. This quickening is attended with a sensation of motion, or fluttering in the lower belly, and is not unfrequently accompanied with faintishness or hysterical irritation." And, again; "when the women have any doubt with regard to their situation, they generally look forward to the end of the fourth month as a period which can ascertain their condition; for at this time, or a little sooner or later in different women, *the motion of the child is first experienced*, *or it is said to* QUICKEN."

The London Practice of Midwifery, Edit. 1811, rumoured* to be a transcript of Dr. Clarke's Lectures, says but little on the subject; and, even in what it does say, blunders egregiously. "The diseases of irritation cease just *before* the time of quickening," is the assertion of this London Practice. Just *after* the time of quickening is the fact: and why this happens can be explained. I must do Dr. Clarke himself the justice, however, to state that this is not his opinion. The complaints of early pregnancy, he says (Essays, &c. 2d edit. 1806), will be relieved by quickening;† but he no where indicates, as far as I know, what the nature of this quickening is. The latest and most respectable Medical Dictionary we have, published in 1809, and which may fairly be admitted to have collected opinions to the period of its publication, when endeavoring to lay down data for regulating the reckoning in pregnancy, says "the whole should be corrected by the *quickening*, *the period when the child's motion is perceived*.‡

From

---

* I think it fair to observe here, that Dr. Clarke has, in the most pointed terms, denied all foundation to this rumour. There is, indeed, an internal evidence in the book itself that will acquit this ingenious teacher. *The mistakes, the flippancy, the pert verbosity, of the London Practice of Midwifery, could never make any part of Dr. Clarke's Lecture.*

† Does the doctor mean to refer here to the motion of the child, or to the change of position in the uterus, under the term quickening. If he refers by this term to the first motions of the child, he is wrong as to the complaints of early pregnancy then ceasing: if he refers by this term to change of position in the uterus, he is wrong in the application of the term, though right in asserting that the complaints of early pregnancy then cease. The change of the position of the uterus is the efficient cause of the cessation of these complaints.

‡ A most respectable Medical Review, having occasion to notice *quickening*, in April 1810, says, "the only physiological question which occurred during the course of this trial (the trial of Pizzy and

 Codd,

From this evidence, it may unquestionably be inferred, that the latest and most esteemed writers on midwifery still hold the opinion that *Quickening* is a peculiar circumstance of the fœtus; and that this circumstance is no other than its first motions consequent on its new mode of existence, which, being felt by the mother, produce the extraordinary and alarming sensation described under the above denomination.

I must reject, however, the received opinion, that the specific sensation experienced by the mother, under the term *Quickening*, is to be ascribed to the first perceptible motion of the fœtus, when that fœtus is vaguely said to become animated, because

1st, The sensation of quickening* is not constant and universal: some women never experience it, others with some of their children only.

2d, It has a distinct character from any subsequent motion of the child: no woman ever admits that it resembles, in the slightest degree, the struggles of the fœtus.

3d, This sensation is never repeated in the same pregnancy, which must happen if it arose from the motion of the child.

4th, It is totally incomprehensible that any motion of which the fœtus is capable, in the fourth month, should

---

Codd, in 1808, for procuring Abortion), and perhaps the only one which properly comes under our consideration, was in regard to the time in which a child quickens. A medical witness, examined, deposed that it took place in about 18 weeks, sometimes at 14, and sometimes not till 20 weeks, but mostly about 18; and he never knew it so late as 25 weeks, though he could not say that it sometimes may not happen until the 21st or 22d. Upon the subject of quickening, we may observe," the reviewer proceeds, "that, although recognised by popular belief, and even by the laws of the land, as something definite, it is not so. The growth and development of the child is gradual and uniformly progressive. During no period of gestation does any sudden revolution or change take place; *and what is called quickening is merely the motions of the child becoming sensible to the mother*."—*Edinburgh Med. and Surg. Jour. April* 1810. 248.

* I do not speak of those at first obscure, but gradually increasing, motions of the child, felt by every pregnant woman, and with numerous repetitions during gestation; but of that singular and specific sensation felt but once in the same pregnancy, always disordering the woman for a time, often occasioning her to faint, and which has been pointed out as marking the period when the embryo receives the principle of animation under the term *quickening*.

communicate communicate such a sensation to the mother as to produce deliquium *animi*.

Considering then that the sensation called *Quickening* is an accidental circumstance, and not depending on any conjectural sudden imparting of life to the embryo, which, if a fact, must be a permanent occurrence; that there is no parity between the effect and the assumed cause; and that this effect, if it arose from any motion of the child, must produce similar sensations in the mother during the subsequent period of gestation, when its struggles are still more palpable; I am compelled to observe, that this opinion is only proper for the dark ages of scholastic confusion, and unsuited to that philosophy which now directs our researches into the laws and operations of Nature.

On the ground above stated, presuming that the opinion which refers the sensation denominated, with gross want of precision, *quickening*, to some sudden imparting of life to the fœtus, or to its trifling and obscure motions when first perceived by the mother, to be altogether untenable; I believe the phenomena, observed without prejudice, will bear me out in the assertion, that it does not arise from the fœtus but from the uterus, and belongs not to the child but to the mother.

It is a known fact that the uterus, in the early period of pregnancy, descends into the pelvis, and there remains, usually, to the latter part of the fourth month; and that, as it enlarges, it necessarily rises above the pelvis into the abdomen. There are three circumstances or facts connected with this change of local position of the uterus.

1st, The ascent of the impregnated uterus from its position in the pelvis to its subsequent station in the abdominal cavity, is sometimes gradual and unobserved: then the sensation called *quickening* is *not* felt.

2d, The uterus is sometimes so impacted in the pelvic cavity, as not to reach its final station within the abdomen, without the assistance of art: then occurs retroverted uterus; during which *quickening* is *never* felt.

3d, At other times, and those frequent though not constant, there exists some slight impediment to the ascent of the uterus; *which being suddenly overcome, it (the uterus) rises at once into the abdominal cavity, constituting what has so long been referred to the fœtus, under the term quickening.*

The sudden intrusion of the volume of the uterus among the abdominal viscera, parts of high sensibility, accompanied with as sudden a removal of pressure from the iliac vessels, is equal to produce the sensation called *quickening*.

No woman *quickens* (I am compelled yet to use this strange term, term, improper when referred to the fœtus, and absurd,* certainly, when applied to the mother,) while the uterus remains in the pelvis, nor when it has risen above the brim of that bony cavity and is settled in its place in the abdomen; *but the sensation is felt in transitu, at the moment when the uterus, removing from the pelvis, enters the abdominal cavity.*

This postulate† admits of verification or rejection by experiment and observation. If those who are extensively employed in the practice of midwifery, will watch the sensation of *Quickening*, simultaneous with the change of locality in the uterus, they will find how near it is to a demonstration.

I am not aware that the specific sensation known so long by the term *quickening*, occurring to *some* pregnant women, has been thus explained before:‡ but I think this explanation removes all difficulty as to the natural fact, as well as it does also the metaphysical jargon about the sudden infusion of the vital principle into the *embryo.*

*London, March* 30, 1812. MEDICUS.

---

* Our best philologists have used the word *quicken* in the absurd sense as applied to the mother. The verb active to quicken is of Saxon etymology, and literally means to *make alive.* The verb neuter to quicken, Johnson explains, *to become alive; as a woman quickens with child:* and he cites passages which prove it can apply only to the embryo.

They rub out of it a red dust, that converteth after a while into worms, which they kill with wine when they begin to *quicken.*
*Sandys.*

These hairs which thou dost ravish from my chin,
Will *quicken* and accuse thee. *Shakespeare.*

† The term *postulate* does not apply to the state of my own mind; but I concede that my position is assumed, until the fact has been examined, and the investigation pursued by others.

‡ That this opinion is not at this moment new to myself, however, I can prove, by a note in the Med. and Phys. Jour. for July 1810, p. 38, where, having occasion to notice *quickening* in consequence of the trial of Pizzy and Codd for procuring abortion, I observed, "quickening, as it is popularly termed, is a sensation of a peculiar quality, occurring to most women at some period of utero gestation, and is believed to be the first indication of life in the fœtus. Perhaps this sensation has no dependance on the life of the fœtus, but is simply produced by the *impregnated uterus rising suddenly out of the pelvis into the cavity of the abdomen.*" More reflection, and additional observation, make me now speak positively to this fact, which, in 1810, I gave to the public with some hesitation.

